# Fixed-bearing unicompartmental vs. total knee arthroplasty in elderly patients with medial osteoarthritis and anterior cruciate ligament deficiency: a retrospective comparative study

**DOI:** 10.3389/fsurg.2026.1866521

**Published:** 2026-08-05

**Authors:** Dongsheng Guo, Xin Li, Wenya Chu, Yuefu Dong, Donglai Wang

**Affiliations:** 1 Department of Orthopedics, Lianyungang Clinical Medical College of Nanjing Medical University, Lianyungang, China; 2 Department of Orthopedics, The Affiliated Suzhou Hospital of Nanjing Medical University, Suzhou, China

**Keywords:** anterior cruciate ligament deficiency, elderly, medial osteoarthritis, total knee arthroplasty, unicompartmental knee arthroplasty

## Abstract

**Background:**

To compare fixed-bearing unicompartmental knee arthroplasty (UKA) and total knee arthroplasty (TKA) in elderly patients with medial compartment osteoarthritis and anterior cruciate ligament (ACL) deficiency.

**Methods:**

A retrospective case-control study with 1:1 propensity score matching was performed, enrolling 85 UKA and 85 TKA patients. Baseline covariates were well balanced (all standardized mean differences <0.1). Perioperative data, knee range of motion (ROM), Western Ontario and McMaster Universities Osteoarthritis Index (WOMAC) scores, Tegner scores, patient satisfaction, and complications were analyzed over 5-year follow-up.

**Results:**

UKA was associated with significantly lower postoperative pain (VAS: 3.65 ± 1.11 vs. 5.03 ± 0.78; MD −1.38, 95% CI −1.67 to −1.09; *P* < 0.0001), hemoglobin loss (8.15 ± 4.32 vs. 10.29 ± 5.88 g/L; MD −2.14, 95% CI −3.69 to −0.59; *P* = 0.0076), and tramadol consumption (774.10 ± 139.90 vs. 1,114.00 ± 308.70 mg; MD −339.90, 95% CI −412.0 to −267.8; *P* < 0.0001), with similar complication rates (2.35% vs. 4.71%; *P* = 0.6819). Postoperative ROM was greater in the UKA group at all time points (1 year: 125.30 ± 9.30° vs. 119.00 ± 10.85°; MD 6.30°, 95% CI 3.26–9.34; *P* < 0.0001). WOMAC scores were lower at 1 year (29.61 ± 12.88 vs. 34.74 ± 12.56; MD −5.13, 95% CI −8.95 to −1.31; *P* = 0.0094) but comparable from 2 to 5 years. At 5 years, instability rates were similar (1.18% vs. 3.53%; *P* = 0.6205), while UKA achieved higher Tegner (4.46 ± 1.14 vs. 3.99 ± 0.78; MD 0.47, 95% CI 0.18–0.76; *P* = 0.0020) and satisfaction scores (7.74 ± 1.10 vs. 6.94 ± 1.44; MD 0.80, 95% CI 0.41–1.19; *P* < 0.0001).

**Conclusion:**

Fixed-bearing UKA may represent a suitable alternative in carefully selected elderly patients with ACL-deficient medial osteoarthritis, yielding favorable perioperative recovery and comparable 5-year safety to TKA. Prospective multicenter studies are required to validate these findings.

## 1 Introduction

Osteoarthritis (OA) is the most common joint disease in the world, affecting approximately 10% of men and 18% of women in the elderly population (1, 2). Approximately 25%–47% of patients with osteoarthritis exhibit degenerative changes localized to the anteromedial compartment of the knee (3, 4). Previous studies have demonstrated that unicompartmental knee arthroplasty (UKA) is associated with less invasiveness, faster recovery, and superior postoperative knee range of motion compared to total knee arthroplasty (TKA) in patients with anteromedial osteoarthritis, supporting its preference in appropriately selected cases (5–7). Nevertheless, elderly patients with osteoarthritis often develop anterior cruciate ligament (ACL) deficiency due to the proliferation of femoral intercondylar osteophyte (8, 9). Studies have shown that in patients with osteoarthritis, 73% exhibit abnormal signals in their ACL (10). Meanwhile, in patients with osteoarthritis combined with ACL deficiency, a large proportion of patients have cartilage damage limited to the medial compartment of the knee (11). In the past, ACL deficiency was often considered a contraindication for UKA (12). For elderly patients with ACL deficiency presenting with isolated medial compartment pain, preserved joint stability, and radiographically confined medial cartilage degeneration, whether UKA should be abandoned in favor of TKA remains controversial despite historical contraindications. Contemporary clinical studies have demonstrated comparable functional outcomes and implant survivorship between UKA patients with ACL deficiency and those with intact ACLs (13–18). Emerging evidence challenges the traditional dogma that an intact ACL is mandatory for UKA. Therefore, we conducted a retrospective study comparing postoperative functional outcomes between UKA and TKA in ACL-deficient elderly patients with isolated medial compartment osteoarthritis.

## 2 Materials and methods

### 2.1 Patient selection

This retrospective analysis included patients undergoing knee arthroplasty at the Joint Center of Lianyungang Clinical College of Nanjing Medical University (December 2015–January 2020). Participants were stratified into two cohorts: UKA group and TKA group.

This study was approved by the Institutional Review Board of the First People's Hospital of Lianyungang in accordance with the Declaration of Helsinki (2013 revision).

Inclusion Criteria: (1) Age ≥70 years; (2) Preoperative MRI-confirmed ACL deficiency (Grades II–III); (3) Medial compartment-localized knee pain; (4) Intact lateral compartment cartilage and meniscus on Magnetic Resonance Imaging(MRI); (5) Knee flexion deformity <15°.

Exclusion Criteria: (1) Incomplete medical records; (2) Traumatic etiology of ACL deficiency (e.g., acute sports injury or accident-related trauma).

ACL deficiencies were classified into three grades based on MRI criteria:

Grade I (Mild): Intrasubstance deficiency without ligament elongation. T1- and T2-weighted sequences demonstrate increased intraligamentous signal intensity, while preserving fiber continuity and maintaining normal ligament morphology (shape, thickness, length).

Grade II (Moderate): Intrasubstance deficiency with ligament elongation. MRI reveals diffuse high signal intensity, ligament thickening (secondary to edema/hemorrhage), and partial fiber discontinuity with irregular contour.

Grade III (Severe): Complete tear characterized by ligament discontinuity, retraction, abnormal orientation, or pseudomass formation, with marked signal hyperintensity across all sequences.

Intraoperative confirmation of ACL status was performed for all patients. The ACL status was evaluated via direct visualization and stress tests. Grade II and Grade III deficiencies were both included in this study, while Grade I deficiencies were excluded. This categorization was based on preliminary biomechanical assessments at our center, which demonstrated that Grade I knees retained near-normal anteroposterior stability, whereas Grade II and Grade III knees exhibited comparable degrees of anterior tibial translation and rotational laxity under manual stress testing. Consequently, Grade II and III were considered functionally equivalent as “ACL-deficient” states in the context of this surgical decision-making analysis, whereas Grade I was considered functionally “ACL-competent” and therefore excluded.

### 2.2 Clinical treatment

TKA was performed with the LEGION™ posterior-stabilized knee system, and UKA with the Fixed-Bearing JOURNEY™ UNI Knee system, both manufactured by Smith & Nephew, Inc. (Andover, Massachusetts, USA). All surgical procedures were executed under general anesthesia by senior orthopedic surgeons with extensive experience in knee arthroplasty. A standard medial parapatellar approach was employed for TKA, with the incision extending 4 cm above the superior pole of the patella to 5 cm below the inferior pole to preserve the structural integrity of the patellar tendon. For UKA, a minimally invasive medial parapatellar approach measuring 8–10 cm in length was adopted to minimize soft tissue trauma and facilitate postoperative recovery. Prosthesis positioning utilized a combined intramedullary and extramedullary alignment system. In TKA, the femoral component was positioned via an intramedullary guide at a valgus angle of 5°–7° relative to the femoral anatomical axis, while the tibial component was aligned with an extramedullary guide to achieve a posterior slope of 3°–5°. For UKA, the femoral medial condyle component was implanted with 3°–5° valgus correction using dedicated distal femoral osteotomy system (spacer block technology), and the tibial component was aligned to restore the native inclination of the medial tibial plateau with a 2°–4° posterior slope via an extramedullary guide. All component positions were verified intraoperatively by fluoroscopy to ensure alignment accuracy.

The choice between UKA and TKA was not randomized but was determined through clinical shared decision-making between the patient and the operating surgeon based on preoperative evaluation. UKA was generally considered for patients who met the following criteria: (1) isolated medial compartment pain localized to the anteromedial region; (2) preserved lateral compartment cartilage and meniscus confirmed on MRI; (3) knee flexion deformity <15°; (4) acceptable coronal alignment (varus deformity generally <10°); and (5) intraoperative confirmation of intact or minimally damaged lateral and patellofemoral cartilage. TKA was preferred for patients with more extensive tricompartmental degenerative changes, significant coronal malalignment beyond the acceptable range for UKA, or patellofemoral symptoms requiring patellar resurfacing. Both senior surgeons (Surgeon A and Surgeon B) were experienced in both procedures and participated in joint decision-making; however, individual surgeon judgment regarding intraoperative bone quality, ligamentous stability, and patient-specific factors (including activity expectations and bone stock preservation) inevitably influenced the final treatment selection. To mitigate potential surgeon-related confounding, surgeon identity was included as a covariate in the propensity score matching model, and the distribution of surgeons was well balanced between the two groups after matching (Table 1).

**Table 1:** Comparison of baseline patient characteristics between the UKA and TKA groups.

Characteristic	UKA (*n* = 85)	TKA (*n* = 85)	Test	*P*
Age	81.61 ± 4.03	82.24 ± 4.27	0.98	0.3291
Gender *n* (%)				
Male	41 (48.24%)	42 (49.41%)	0.15	0.8781
Female	44 (51.76%)	43 (50.59%)		
Affected side, *n* (%)				
Left	38 (44.71%)	40 (47.06%)	0.31	0.7582
Right	47 (55.29%)	45 (52.94%)		
BMI	23.26 ± 3.43	23.22 ± 3.36	0.08	0.9353
ACL deficiency grade, *n* (%)				
Grade II	21 (24.71%)	23 (27.06%)	0.35	0.7262
Grade III	64 (75.29%)	62 (72.94%)		
OA severity (Kellgren–Lawrence grade), *n* (%)				
III	36 (42.35%)	33 (38.82%)	0.47	0.6394
IV	49 (57.65%)	52 (61.18%)		
Comorbidity burden	8.13 ± 3.60	8.26 ± 3.45	0.24	0.8113
Patellofemoral joint status, *n* (%)				
No patellofemoral arthritis	14 (16.47%)	10 (11.76%)	0.88	0.3783
With patellofemoral arthritis	71 (83.53%)	75 (88.24%)		
Operating surgeon, *n* (%)				
Surgeon A	45 (52.94%)	47 (55.29%)	0.31	0.7582
Surgeon B	40 (47.06%)	38 (44.71%)		
Year of surgery, *n* (%)				
2015–2017	29 (34.12%)	31 (36.47%)	0.32	0.7482
2018–2020	56 (65.88%)	54 (63.53%)		
Preoperative WOMAC score	71.08 ± 6.95	73.16 ± 7.80	1.84	0.0667
Preoperative knee range of motion	112.40 ± 14.50	111.40 ± 11.30	0.55	0.5838

Intraoperative bleeding control was achieved through a pneumatic tourniquet applied to the upper thigh. Controlled hypotensive anesthesia was administered concurrently to maintain a mean arterial pressure of 60–70 mmHg, further reducing intraoperative blood loss. Postoperatively, anticoagulation prophylaxis was administered with rivaroxaban at a dosage of 10 mg once daily for 14 days. Severe postoperative pain was managed with oral tramadol, prescribed as 50–100 mg per dose as needed, with a maximum daily dosage not exceeding 400 mg. Early functional rehabilitation, encompassing passive and active range-of-motion exercises, was initiated within 24 h postoperatively to optimize joint function recovery. Patients were discharged approximately 1 week after surgery and scheduled for follow-up evaluations at 1, 3, and 6 months postoperatively, followed by annual assessments thereafter to monitor long-term outcomes.

### 2.3 Clinical evaluation

The following parameters were systematically documented:

1. Baseline Characteristics: Age, gender, affected side, body mass index (BMI), ACL deficiency grades, OA severity (Kellgren–Lawrence grade), preoperative Western Ontario and McMaster Universities Osteoarthritis Index (WOMAC) score, and preoperative knee range of motion (ROM), comorbidity burden (Charlson Comorbidity Index), patellofemoral joint status (Concurrent patellofemoral arthritis status), operating surgeon, and year of surgery. Comorbidity burden was quantified using the Charlson Comorbidity Index (CCI) version 1.3 based on ICD-10 codes from electronic health records. Each condition was assigned a weight (1, 2, 3, or 6) according to the prognostic risk. Age adjustment was performed with +1 point for 50–59 years, +2 for 60–69, +3 for 70–79, and +4 for ≥80 years.
2. Perioperative Metrics: Postoperative pain was assessed using the Visual Analog Scale (VAS) score at 7 days, hemoglobin loss was calculated as the admission-to-discharge hemoglobin difference from routine blood tests, analgesic requirement was measured as total postoperative tramadol consumption, and perioperative complications were collected.
3. Postoperative Functional Recovery: Knee ROM was measured postoperatively at 7 days, 1 month, 3 months, and 1 year, and WOMAC scores were assessed annually from Year 1 to 5.
4. Long-Term Outcomes (5-year follow-up): Ligament-related complications including instability and dislocation, Tegner activity level score at 5 years and patient satisfaction were evaluated. Patient satisfaction was evaluated with a 10-point numerical rating scale (0–10, 10 = complete satisfaction).

### 2.4 Statistical analysis

The sample size was calculated using G*Power 3.1 software (version 3.1.9.7 Heinrich Heine University, Düsseldorf, Germany). Assuming a significance level (*α*) of 0.05, statistical power (1-*β*) of 80%, and a medium effect size (Cohen's *d* = 0.5) based on previous literature comparing UKA and TKA outcomes, the minimum required sample size was estimated to be 148 patients to detect a clinically meaningful difference.

Propensity score matching (PSM) was performed to reduce confounding bias. The propensity score was estimated using a logistic regression model with group as the dependent variable and the following covariates: age, gender, affected side, BMI, ACL deficiency grade, OA severity, preoperative WOMAC score, preoperative ROM, comorbidity burden, patellofemoral joint status, operating surgeon, and year of surgery. A 1:1 nearest-neighbor matching was implemented without replacement. Balance diagnostics were assessed using standardized mean differences (SMD) for all covariates entered into the propensity score model. SMD values before and after matching are reported in Supplementary Table 1. A post-matching SMD <0.1 was considered indicative of adequate balance.

Before statistical tests, the normality of continuous data was verified using the Kolmogorov–Smirnov test, and homogeneity of variance was tested via the Levene test.

Continuous variables are presented as mean ± standard deviation (SD) and compared using Student's *t*-test. Categorical variables were presented as number (percentage, %). Pearson's chi-square test was used for intergroup comparisons when the expected frequency of all cells in the contingency table was ≥5. For categorical data with an expected frequency <5 in any cell, or for rare events with extremely low incidence, Fisher's exact test was applied for group comparisons. While non-normally distributed data were analyzed with nonparametric tests. Repeated-measures analysis of variance (ANOVA) was used to analyze ROM and WOMAC scores measured at multiple time points. The main effects of time, group, and time × group interaction were assessed. Mauchly's test was used to verify sphericity, with Greenhouse–Geisser correction applied if violated. Independent *t*-tests with Bonferroni correction were used for intergroup comparisons at each time point. Statistical significance was defined as *P* < 0.05. No missing data were present for the variables analyzed in this study. All continuous and categorical variables were complete for the 170 included patients. Consequently, complete case analysis was performed without the need for missing data imputation. All analyses were conducted in IBM SPSS Statistics (version 23.0; IBM, NY, USA).

## 3 Results

### 3.1 Baseline characteristics of patients

**Figure 1:**
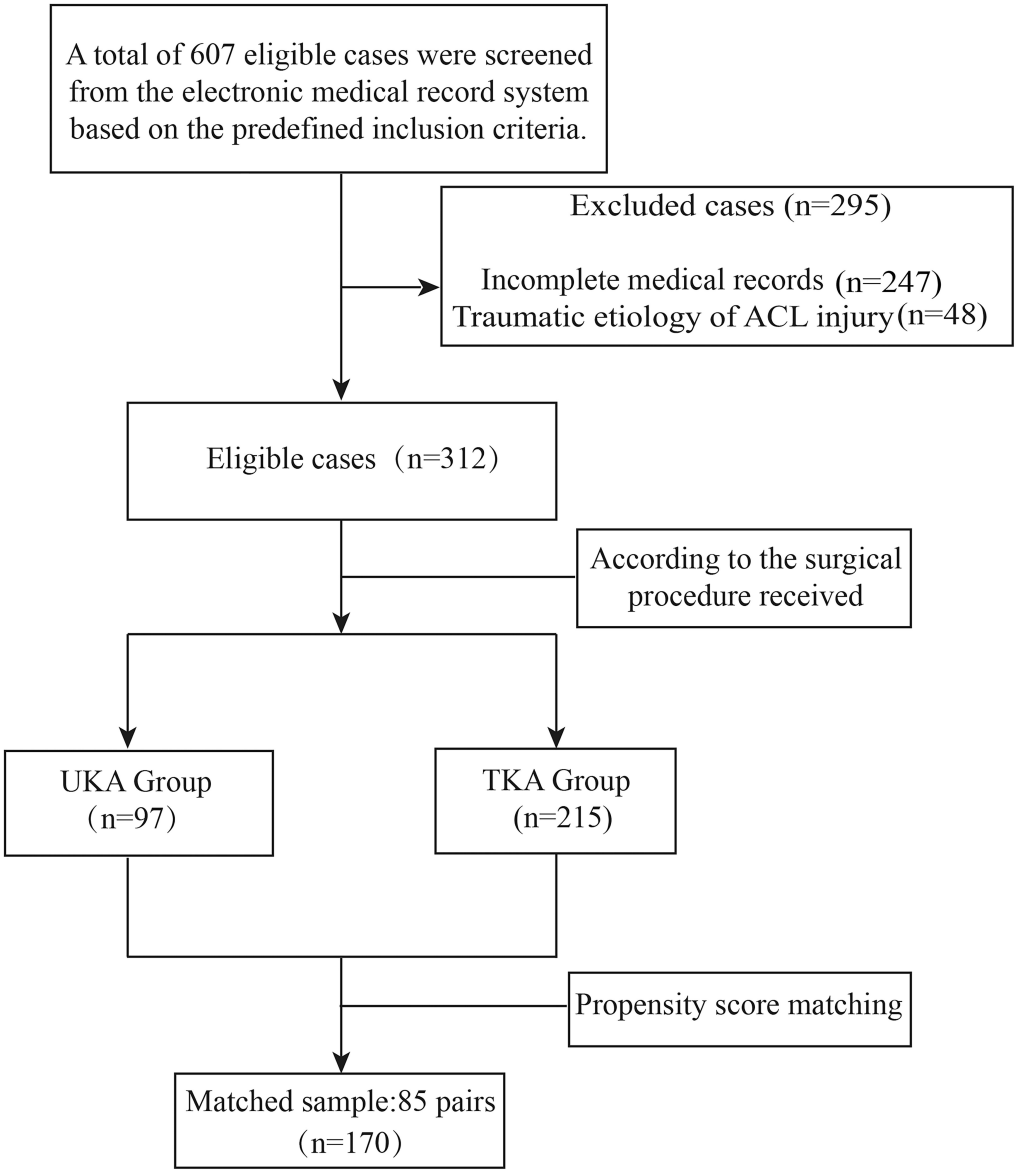
Patient selection flowchart.

The study initially enrolled 607 eligible patients. After applying the exclusion criteria, 97 patients remained in the UKA group and 215 in the TKA group. Following PSM, 170 patients were enrolled, including 85 in the UKA group and 85 in the TKA group (Figure 1). The sample size exceeded the required minimum of 148. There were no significant differences in age, gender, affected side, BMI, ACL deficiency grade, OA severity, preoperative WOMAC score, preoperative ROM, comorbidity burden, patellofemoral joint status, operating surgeon, and year of surgery (Table 1). All standardized mean differences after propensity score matching were <0.1 (Supplementary Table 1), confirming adequate covariate balance and supporting the validity of subsequent between-group comparisons.

### 3.2 Perioperative outcomes and early functional recovery

Compared with the TKA group, the UKA group had significantly lower VAS pain score at 1 week postoperatively (3.65 ± 1.11 vs. 5.03 ± 0.78, *P* < 0.05), less hemoglobin loss (8.15 ± 4.32 vs. 10.29 ± 5.88 g/L, *P* < 0.05), and lower total tramadol consumption (774.10 ± 139.90 vs. 1,114.00 ± 308.70 mg, *P* < 0.05). The incidence of perioperative complications was comparable between the two groups (2.35% vs. 4.71%, *P* > 0.05) (Table 2). Preoperative knee range of motion was similar between the UKA and TKA groups (112.40 ± 14.50 vs. 111.40 ± 11.30°, *P* > 0.05). Repeated-measures ANOVA showed significant main effects of time, group, and a significant time × group interaction (*P* < 0.05, Supplementary Table 2). *Post hoc* comparisons revealed that the UKA group had significantly greater ROM than the TKA group at 7 days, 1 month, 3 months, and 1 year postoperatively (*P* < 0.05) (Table 3).

**Table 2:** Comparison of perioperative conditions between UKA and TKA groups.

Group/Statistic	VAS score 1 week postop	Haemoglobin loss (g/L)	Total tramadol usage (mg)	Perioperative complications (%)
UKA	3.65 ± 1.11	8.15 ± 4.32	774.10 ± 139.90	2.35%
TKA	5.03 ± 0.78	10.29 ± 5.88	1,114.00 ± 308.70	4.71%
Test value	9.443	2.703	9.25	–
*P*	<0.0001	0.0076	<0.0001	0.6819
Mean difference/Risk difference (95% CI)	−1.38 (−1.67, −1.09)	−2.14 (−3.69, −0.59)	−339.90 (−412.0, −267.8)	−2.36% (−7.90%, 3.18%)
Effect size	Cohen's *d* = 1.44	Cohen's *d* = 0.42	Cohen's *d* = 1.42	Cramer’s *V* = 0.06

The normality of continuous variables was verified using the Kolmogorov–Smirnov test, and all were approximately normally distributed (*P* > 0.05).

**Table 3:** Comparison of preoperative and postoperative knee range of motion between UKA and TKA groups.

Range of motion (°)	Preoperatively	Postoperatively			
		7 days	1 month	3 months	1 year
UKA	112.40 ± 14.50	98.40 ± 5.33	112.00 ± 13.50	121.10 ± 12.19	125.30 ± 9.30
TKA	111.40 ± 11.30	89.72 ± 8.65	106.70 ± 12.76	112.4 ± 12.35	119.00 ± 10.85
Test value	0.55	7.88	2.62	4.66	4.04
*P*	0.5838	<0.0001	0.0097	<0.0001	<0.0001
Mean difference (95% CI)	1.00 (−2.91, 4.91)	8.68 (6.52, 10.84)	5.30 (1.35, 9.25)	8.70 (5.01, 12.39)	6.30 (3.26, 9.34)
Cohen's *d*	0.08	1.21	0.4	0.71	0.62

Repeated-measures ANOVA showed significant main effects of time, group, and a significant time × group interaction (*P* < 0.05, Supplementary Table 2).

### 3.3 Postoperative functional outcomes and 5-year long-term complications

Preoperative WOMAC scores were comparable between the UKA and TKA groups (*P* > 0.05). Repeated-measures ANOVA showed significant main effects of time and a significant time × group interaction (*P* < 0.05, Supplementary Table 2). *Post hoc* comparisons demonstrated that the UKA group had significantly lower WOMAC scores than the TKA group at 1 year postoperatively (*P* < 0.05), while no significant differences were found at 2, 3, 4, and 5 years postoperatively (*P* > 0.05) (Table 4).

**Table 4:** Comparison of preoperative and 1- to 5-year postoperative WOMAC scores between UKA and TKA groups.

WOMAC score	Preoperatively	Postoperatively				
		1 year	2 years	3 years	4 years	5 years
UKA	71.08 ± 6.95	29.61 ± 12.88	17.78 ± 7.98	16.72 ± 7.65	17.73 ± 7.18	18.31 ± 7.90
TKA	73.16 ± 7.80	34.74 ± 12.56	18.73 ± 7.81	16.31 ± 7.84	17.88 ± 8.33	16.89 ± 6.79
Test value	1.84	2.63	0.79	0.35	0.13	1.25
*P*	0.0667	0.0094	0.4323	0.7293	0.8981	0.2133
Mean difference (95% CI)	−2.08 (−4.30, 0.14)	−5.13 (−8.95, −1.31)	−0.95 (−3.32, 1.42)	0.41 (−1.92, 2.74)	−0.15 (−2.49, 2.19)	1.42 (−0.80, 3.63)
Cohen's *d*	0.28	0.4	0.12	0.05	0.02	0.19

Repeated-measures ANOVA showed significant main effects of time and a significant time × group interaction (*P* < 0.05).

At the 5-year follow-up, the incidence of instability and dislocation was low and comparable between groups (*P* > 0.05). Patients in the UKA group achieved significantly higher Tegner scores (4.46 ± 1.14 vs. 3.99 ± 0.78, *P* < 0.05) and higher satisfaction scores (7.74 ± 1.10 vs. 6.94 ± 1.44, *P* < 0.05) compared with the TKA group (Table 5).

**Table 5:** Five-year long-term outcomes, complications.

Group/Statistic	Instability	Dislocation	Tegner score	Patient satisfaction score
UKA	1	0	4.46 ± 1.14	7.74 ± 1.10
TKA	3	0	3.99 ± 0.78	6.94 ± 1.44
Test value	–	–	3.14	4.07
*P*	0.6205	–	0.0020	<0.0001
Mean difference/Risk difference (95% CI)	−2.35% (−8.9%, 4.2%)		0.47 (0.18, 0.76)	0.80 (0.41, 1.19)
Effect size	Cramer's *V* = 0.04		Cohen's *d* = 0.48	Cohen's *d* = 0.63

Instability was compared using the Fisher exact test.

### 3.4 Representative case

**Figure 2:**
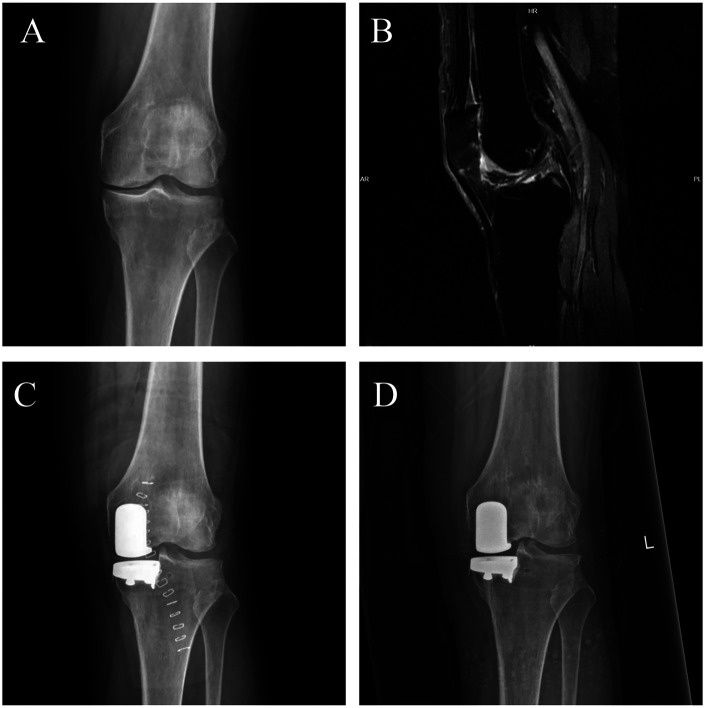
Representative case. **(A)** Preoperative radiograph showing medial joint space narrowing, subchondral sclerosis, and Kellgren–Lawrence grade III osteoarthritis. **(B)** Sagittal T2-weighted MRI demonstrating ACL elongation with partial fiber discontinuity, consistent with Grade II deficiency. **(C)** Postoperative radiograph at day 1 showing well-positioned fixed-bearing UKA components. **(D)** Five-year follow-up radiograph demonstrating maintained component alignment without loosening, subsidence, or lateral compartment progression.

An 83-year-old female patient presented with isolated pain localized to the medial compartment of the left knee (Figure 2). Preoperative MRI confirmed Grade II ACL deficiency, intact cartilage and meniscus in the lateral compartment, and K-L Grade III osteoarthritis in the medial compartment. The patient met all inclusion criteria: (1) age ≥70 years; (2) MRI-confirmed ACL deficiency (Grades II–III); (3) pain localized to the medial compartment of the knee; (4) intact lateral compartment structures; and (5) knee flexion deformity <15°. Intraoperative exploration confirmed partial fiber discontinuity of the ACL, and the anterior drawer test was positive. She underwent fixed-bearing UKA. At 5-year follow-up, her WOMAC score was 19, Tegner activity score was 5, and patient satisfaction score was 8, with no evidence of instability or dislocation.

## 4 Discussion

This retrospective case-control study using PSM compared the mid-term outcomes of fixed-bearing UKA vs. TKA in elderly patients with medial compartment knee osteoarthritis and ACL deficiency. The primary finding is that in carefully selected elderly patients with ACL-deficient medial osteoarthritis who maintain functional knee stability, fixed-bearing UKA may be considered a viable alternative to TKA. UKA provided comparable mid-term safety and stability, along with favorable perioperative recovery, early functional outcomes, and higher patient satisfaction compared to TKA.

UKA was associated with reduced perioperative trauma, milder postoperative pain, less blood loss, and lower analgesic consumption, with complication rates comparable to TKA. This aligns with previous research findings (19, 20). As a minimally invasive procedure, UKA preserves bone stock and soft tissue structures, which aligns with the physiological status of elderly patients and facilitates rapid postoperative rehabilitation. Functionally, UKA yielded superior knee ROM at all follow-up time points and lower 1-year WOMAC scores, with comparable functional outcomes between the two groups at 2–5 years postoperatively. Mid-term follow-up demonstrated similar low rates of joint instability and dislocation between groups, while UKA was associated with significantly higher Tegner activity scores and patient satisfaction. The inherently lower mechanical demand of elderly patients correlates with reduced polyethylene wear rates compared to younger cohorts (21, 22). This biomechanical profile, combined with the priority of minimizing perioperative risks in this demographic, supports the choice of a tissue-preserving strategy like UKA.

The positive outcomes of this study are specific to fixed-bearing UKA systems. This warrants an in-depth discussion of the implant design rationale. In our clinical practice, we have observed that ACL deficiency leads to significant kinematic alterations in the mobile-bearing insert. Notably, to maintain joint stability in patients with ACL insufficiency, mobile-bearing UKA often necessitates the implantation of a thicker polyethylene insert. This compensatory increase in insert thickness, however, may elevate the contact pressure within the lateral compartment, potentially accelerating cartilage wear in that region. The fixed-bearing knee system was employed in the present study specifically because its design mitigates insert kinematic variability and helps prevent excessive valgus alignment, which is theorized to offer a more stable environment in the ACL-deficient knee. The Fixed-Bearing Knee system was chosen for this study, which by design eliminates the occurrence of insert trajectory changes and excessive valgus.

Theoretically, the Fixed-Bearing UKA system is more suitable for patients with ACL deficiencies (23). Some scholars believe that such patients can be treated through a combination of UKA and ACL reconstruction (8, 24, 25). However, there are certain drawbacks, such as delayed rehabilitation increasing the risk of knee stiffness, and technical challenges for future revision if needed. Our results demonstrate that isolated fixed-bearing UKA can provide clinically acceptable mid-term joint stability—defined by the absence of symptomatic instability, dislocation, or revision—and is well adapted to the biomechanical environment of ACL-deficient knees in the elderly. However, we acknowledge that this conclusion is based on clinical functional outcomes and complication rates, not on radiographic evidence of implant fixation, alignment maintenance, or lateral compartment preservation. Fixed-bearing UKA provided comparable mid-term clinical safety and symptomatic joint stability to TKA over the 5-year follow-up period, with zero revisions observed in both groups. However, this absence of revision events precludes any assessment of long-term implant survivorship, and the 5-year horizon is insufficient to exclude late mechanical failure or polyethylene wear.

This conclusion is supported by other studies. Kikuchi et al. concluded through a retrospective study that ACL deficiency is not always a contraindication for medial UKA in patients with typical anteromedial osteoarthritis radiographs (15). Genfa Du et al. reached a similar conclusion through a systematic review and meta-analysis (14). It is crucial to emphasize the specific population of this study: elderly patients. Our center has not utilized UKA in younger patients presenting with medial osteoarthritis and co-existing ACL deficiencies, as existing studies indicate higher revision rates with UKA than TKA in such cases (5, 6). For younger patients, we are reluctant to take the risk of attempting UKA, as it may potentially lead to increased medical expenses and surgical trauma for the patients in the future.

This study originated from clinical practice. It focused on elderly patients who initially showed clinical symptoms suggesting they might be candidates for UKA. However, subsequent evaluations after seeking medical help confirmed they had ACL deficiencies. This situation posed a crucial question for physicians: whether to choose TKA or UKA for these patients. In our research, we compared the outcomes of TKA and UKA. The results indicated that, even in the presence of ACL deficiencies, UKA led to better clinical outcomes than TKA. This finding has significant practical implications for clinical decision-making in orthopedic surgery.

Population-level registries consistently report higher revision rates for UKA than TKA (26). A worldwide meta-analysis reported 15-year pooled revision rates of 11.04% vs. 5.63% for TKA (27). Nordic registries have further demonstrated 10–15-year UKA survivorship of 69%–84% compared with over 88% for TKA (28). Our zero-revision finding at 5 years likely reflects strict patient selection involving elderly patients with fixed-bearing implants and intact lateral compartments, coupled with lower activity demands in this demographic. These favorable mid-term results should not be interpreted as refutation of established registry evidence, and longer follow-up is required to assess true implant survivorship in this specific population.

Several limitations of this study warrant acknowledgment. First, this single-center retrospective design carries inherent selection bias, as the choice between UKA and TKA was not randomized and likely depended on surgeon preference and individual patient characteristics. Although 1:1 propensity score matching effectively balanced the measured baseline covariates, several important unmeasured confounders could not be fully adjusted for. These include preoperative activity level, lower limb alignment severity, postoperative rehabilitation adherence, socioeconomic factors, and preoperative patient expectations regarding surgical outcomes and desired activity levels. These unmeasured variables may have influenced both the treatment selection and subsequent functional outcomes, and their absence from the propensity score model represents a limitation of our analysis. Consequently, the observed differences between groups should be interpreted as associations rather than definitive causal effects, and readers should exercise caution when generalizing these findings to broader populations. Second, while the sample size meets the pre-specified requirement for the primary endpoint, it has insufficient statistical power for rare low-incidence events, such as prosthesis revision and severe perioperative complications, limiting the robustness of comparative safety analyses between the two procedures. Third, the 5-year follow-up only reflects mid-term outcomes, and is inadequate to fully evaluate long-term implant durability, including late-onset prosthesis loosening and polyethylene wear. Finally, the absence of subgroup analyses stratified by ACL deficiency grade and serial radiographic assessments restricts in-depth mechanistic interpretation of implant stability in ACL-deficient knees. Future multicenter studies with larger cohorts, longer follow-up, and detailed radiographic evaluations are needed to validate these findings and compare outcomes between partial and complete ACL deficiency.

## 5 Conclusion

In carefully selected elderly patients with medial compartment osteoarthritis and ACL deficiency, fixed-bearing UKA may be a viable option with favorable perioperative recovery and improved activity, and comparable 5-year safety to TKA. These single-center, retrospective findings require prospective multicenter validation.

## Data Availability

The raw data supporting the conclusions of this article will be made available by the authors, without undue reservation.

## Supplementary material

Supplementary Table 1 Standardized mean differences (SMDs) of baseline covariates before and after propensity score matching.

Supplementary Table 2 Results of repeated-measures ANOVA for knee range of motion (ROM) and WOMAC scores.
