# Supplementary material for: Fixed-bearing unicompartmental vs. total knee arthroplasty in elderly patients with medial osteoarthritis and anterior cruciate ligament deficiency: a retrospective comparative study
Source: Front Surg. 2026 Aug 5;13:1866521. doi: 10.3389/fsurg.2026.1866521 (PMC13485871; doi:10.3389/fsurg.2026.1866521)
Supplement: Supplementary file 1 [file Table1.docx]

Supplementary Table 1. Standardized Mean Differences (SMD) of Baseline Covariates Before and After Propensity Score Matching

| Covariate | Variable Type | Before PSM | After PSM |
| --- | --- | --- | --- |
| Age | Continuous | 0.152 | 0.028 |
| Gender (Male) | Binary | 0.023 | 0.015 |
| Affected side (Left) | Binary | 0.047 | 0.012 |
| BMI | Continuous | 0.012 | 0.008 |
| ACL deficiency grade (Grade II) | Binary | 0.054 | 0.022 |
| OA severity (Kellgren-Lawrence Grade III) | Binary | 0.072 | 0.035 |
| Comorbidity burden (CCI) | Continuous | 0.037 | 0.015 |
| Patellofemoral joint status (No arthritis) | Binary | 0.136 | 0.078 |
| Operating surgeon (Surgeon A) | Binary | 0.047 | 0.018 |
| Year of surgery (2015–2017) | Binary | 0.049 | 0.025 |
| Preoperative WOMAC score | Continuous | 0.282 | 0.055 |
| Preoperative knee range of motion | Continuous | 0.077 | 0.02 |
