# Supplementary material for: Fixed-bearing unicompartmental vs. total knee arthroplasty in elderly patients with medial osteoarthritis and anterior cruciate ligament deficiency: a retrospective comparative study
Source: Front Surg. 2026 Aug 5;13:1866521. doi: 10.3389/fsurg.2026.1866521 (PMC13485871; doi:10.3389/fsurg.2026.1866521)
Supplement: Supplementary file 2 [file Table2.docx]

Supplementary Table 2. Results of Repeated-Measures ANOVA for Knee Range of Motion (ROM) and WOMAC Scores

| Outcome | Effect | df | F | *P* -value | partial η² |
| --- | --- | --- | --- | --- | --- |
| ROM | Time | 2.84, 477.1 | 286.35 | <.0001 | 0.63 |
|  | Group | 1, 168 | 18.56 | <.0001 | 0.1 |
|  | Time × Group | 2.84, 477.1 | 8.42 | <.0001 | 0.05 |
| WOMAC | Time | 3.41, 573.1 | 308.72 | <.0001 | 0.64 |
|  | Group | 1, 168 | 3.08 | 0.081 | 0.02 |
|  | Time × Group | 3.41, 573.1 | 4.28 | 0.003 | 0.03 |
